# Aptamers as biomarkers for neurological disorders. Proof of concept in transgenic mice

**DOI:** 10.1371/journal.pone.0190212

**Published:** 2018-01-05

**Authors:** Soizic Lecocq, Katia Spinella, Bruno Dubois, Simone Lista, Harald Hampel, Gregory Penner

**Affiliations:** 1 NeoNeuro SAS, Paris, France; 2 Centre des Maladies Cognitives et Comportementales (IM2A), Pavillon François Lhermitte, Hôpital University of Pitié Salpêtrière, Paris France; 3 Institut du Cerveau et de la Moelle épinière (ICM), Hôpital Pitié Salpêtrière, Paris, France; 4 UMR-S975, École des Neurosciences, Paris, France; 5 Université Pierre et Marie Curie-Paris 6, Paris, France; 6 AP-HP, Paris, France; 7 AXA Research Fund & UPMC Chair, Sorbonne Universités, Paris, France; 8 Inserm, Paris, France; 9 CNRS, Paris, France; 10 Département de Neurologie, Institut de la Mémoire et de la Maladie d’Alzheimer (IM2A), Hôpital Pitié-Salpêtrière, Paris, France; Consiglio Nazionale delle Ricerche, ITALY

## Abstract

The act of selecting aptamers against blood serum leads to deep libraries of oligonucleotide sequences that bind to a range of epitopes in blood. In this study we developed an enriched aptamer library by performing positive selection against a pool of blood serum samples from transgenic mice (P301S) carrying the human tau gene and counter selecting against pooled blood serum from negative segregant (wild type) mice. We demonstrated that a large proportion of the aptamer sequences observed with next generation sequence (NGS) analysis were the same from selection round 5 and selection round 6. As a second step, we applied aliquots of the selection round 5 enriched library to blood serum from 16 individual mice for a single round of selection. Each of these individual libraries were characterized through NGS analysis and the changes in relative frequency in aptamers from transgenic mice versus wild type were used to construct a diagnostic fingerprint of the effect of the action of the transgene on the composition of blood serum. This study serves as a model for similar applications with human subjects.

## Introduction

Alzheimer’s Disease (AD) is one of the most common form of dementia occurring in the elderly population worldwide. The main symptoms include memory loss, cognitive impairment, disorientation, and psychiatric symptoms [1,2]. The preliminary diagnosis of AD is made by a combination of clinical criteria which includes a neurological examination, mental status tests and brain imaging [3]. The development of effective treatments for any disease necessarily starts with an accurate diagnosis of the disease in affected patients. It is necessary to know which patients are affected with the same pathology and which ones are not. The definition of AD remains based on autopsy analysis of brain tissue and the direct observation of tau tangles and beta-amyloid fibrils as defined by Alzheimer. Several means of diagnosing the presence and severity of the disease have been developed for living patients including positron emission tomography (PET) scan probes that detect the accumulation of amyloid, and the quantitative presence of protein and peptide biomarkers in cerebrospinal fluid (CSF). PET scan analysis is time consuming and expensive, and many patients are reluctant to provide CSF due to the discomfort associated with the extraction of the fluid and the potential risks for infection. At present there are no clinically validated diagnostic tests for AD in more readily available biological fluids such as blood, urine or saliva. As such, advances in guiding the care of patients, and the development of therapies for this disorder are presently constrained by diagnostic capacity [4, 5, 6, 7].

The World Health Organization (WHO) defines a biomarker as any substance, structure, or process that can be measured in the body or its products and influence or predict the incidence of outcome or disease” [2]. A key difficulty associated with the characterization of biomarkers for neurological disorders is the nature of transmission of pathological signatures on the molecular level through the brain blood barrier (BBB). Proteins are the dominant biomarker target based on immunological detection and as such are the basis for most medical diagnostic tests. Proteins however, are generally cleaved into relatively small peptides for BBB clearance, and these peptides are difficult to characterize using proteomic platforms such as LC/MS-MS. Metabolites, especially cholesterol derivatives, represent another possible source of biomarkers in blood for neurological disorders but these molecules tend to be hydrophobic and as such are predominantly present in blood, aspecifically bound to blood proteins such as serum albumin.

We suggest that the biomarkers for neurological disorders such as Alzheimer's disease may be present in blood but that our present toolkit may not be well suited for their identification and characterization.

In this paper we present an approach to overcome this difficulty. NeoVentures Biotechnology Inc. has developed an aptamer selection platform that does not require immobilization of the target material called FRELEX (WO 2017035666 A1). This platform enables the selection of aptamer libraries against bodily fluids without pre-existing knowledge of the targets being selected for. We developed an enriched aptamer library by performing positive aptamer selection against blood serum pooled from transgenic mice carrying a human tau gene, with counter selection against a pool of blood serum from wild type mice. Through the use of next generation sequencing (NGS) we observed that the majority of the enriched aptamer sequences were observed in both selection round 5 and selection round 6.We applied aliquots of the selection round 5 enriched library to blood serum from 16 mice for a single round of individual selection. Each of these individual libraries were characterized by NGS analysis and we were able to characterize clear differences between late stage transgenic mice and wild type mice in terms of differences in the relative abundance of certain aptamers.

Our hypothesis was that the relative frequency of aptamers within a selected library are a function of the binding affinity of each aptamer for an epitope and the relative concentration of the epitopes that the aptamers bind to. A logical consequence of this hypothesis is that if aliquots from the same selected aptamer library are applied to different samples for a single selection round, then the only reason for differences in the observed frequency of aptamers after such a selection would be differences in the frequency of epitopes that the aptamers bind to within these samples. If this conjecture is valid then it should be possible to use the relative frequency of aptamers within such selected libraries across individual samples to diagnose a phenotypic difference among the individuals from which the samples were derived. In this study we confirmed that this hypothesis was valid for the differentiation of transgenic (human tau) mice from wild type mice. The validation of this hypothesis provides us with a basis to apply this approach to the analysis of similar phenotypes in blood serum from human subjects.

## Materials and methods

### Preparation of DNA library and primers

The DNA library for selection was composed of a 40-mer random region flanked by two constant regions for primer hybridization: 5′-TAG GGA AGA GAA GGA CAT ATG AT-(N40)-TTG ACT AGT ACA TGA CCA CTT GA-3′ (Trilink Biotechnologies, San Diego, CA, USA). Primers used for amplification were purchased from IDT DNA technologies.

### Animals and blood sampling

Transgenic mice used in this study were B6; C3-Tg (Prnp-MAPT*P301S) PS19Vle/J mice (P301S) expressing P301S human tau T34 isoform (1N4R) under the control of PrP promoter. Mice were obtained from The Jackson Laboratory (stock #008169). NeoNeuro has developed their own breeding colony of these mice by crossing hemizygous males with homozygous negative segregating females. Progeny were genotyped from toe extracted DNA using the following primers: forward, 5'- GGC ATC TCA GCA ATG TCT CC-3'; and reverse, 5'- GGT ATT AGC CTA TGG GGG ACA C-3'. All animal procedures and experimental protocols were approved by the Institut du Cerveau et de la Moelle Épinière with the approval of the French National Ethics Committee (Comité National de Réflexion Éthique sur l’Éxpérimentation Animale; Ce5/2011/056) in accordance with European regulations.

Blood was collected from the tail vein or after euthanasia from mice at various ages. Blood was collected into tubes without anticoagulant, allowed to clot for at least 30 min, centrifuged for 10 min at 2000 g and 5 min at 10 000 g. Serum was subsequently removed and frozen (–20°C) before analysis. Mice were humanely sacrificed by cervical dislocation.

### Development of an enriched aptamer library

#### Principle of FRELEX

**Fig 1:**
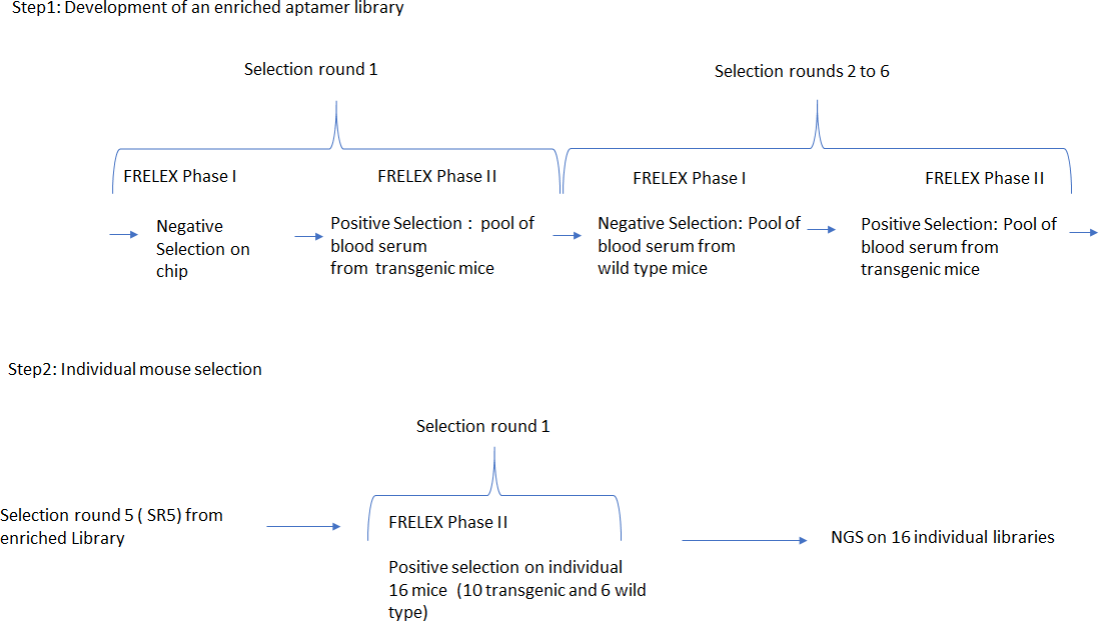
Experimental design.

FRELEX is a method for aptamer selection developed by NeoVentures Biotechnology Inc. (Fig 1) involving the non-immobilization of the target molecule. This allows for a true free-free selection strategy. FRELEX is composed of two phases: for Selection round 1(SR1), Phase I, a DNA aptamer library in solution is added to the random 8mers immobilized through thiol bonds to a gold surface, called the immobilization field. Aptamer sequences with completely closed structures that do not bind to the chip are discarded. Aptamers bound to the 8mers are eluted and purified for the next positive selection. For selection round 2 and onwards (SR2-6), Phase I involves a negative selection in the presence of a pool of healthy blood serum. This means that aptamers that bind to targets within the healthy blood serum are not able to bind to the 8mers on the surface and are discarded. Phase II of FRELEX, the aptamer library from Phase 1 is incubated with a pool of blood serum from transgenic mice for positive selection. We retain only those aptamers that do not bind to the 8mers as their binding to the target inhibits their capacity to bind to the 8mers.

#### Experimental methods

NeoNeuro SAS was kindly permitted to use the FRELEX selection platform (patent application: WO 2017035666 A1) for this study by NeoVentures Biotechnology Inc. for the selection of aptamer libraries. FRELEX requires the preparation of an immobilization field consisting of a gold chip coated with thiolated random 8 base pair DNA oligonucleotides. The 8-mer thiolated random oligonucleotides were dissolved in 50 μL of phosphate buffer saline (PBS) (8.0 mM Na_2_PO_4_, 1.4 mM KH_2_PO_4_, 136 mM NaCl, 2.7 mM KCl, pH 7.4) at a concentration of 10 μM. This solution was incubated at room temperature (RT) for 1 hour on gold surface chip, dimensions 7 x 10 x 0.3 mm (Xantec, Germany). The chip was then air-dried and 50 μL of a solution containing thiol terminated polyethylene glycol (SH-PEG) molecules and incubated for 30 min at RT with gentle shaking. This step blocks any remaining gold surface that is not covered with 8mers. SH-PEG was subsequently added a second time for 16 hours. After that, the SH-PEG solution was removed from the chip and the functionalized gold chip surface was washed with de-ionised water and air-dried.

In the first step of FRELEX employed in this study, 10^15^ sequences from the random aptamer library described previously were snap cooled by heating the library to 95°C for 10 min followed by immediate immersion in ice bath. These single stranded (ss) DNA sequences were incubated with the functionalized immobilization field (gold chip with 8mers) in 50 μL of Selection Buffer (10 mM HEPES, 120 mM NaCl, 5 mM MgCl_2_, 5 mM KCl) for 30 min at RT. The remaining solution was removed and discarded. This removes sequences that have too much secondary structure to enable binding to the 8mers on the surface. The immobilization field was washed twice with 50 μL of 10X TE buffer and the remaining bound oligonucleotides were eluted and recovered with two incubations of 15 min each 1 mL of Selection Buffer at 95°C. These elutions were pooled and purified using the GeneJET PCR Purification Kit (Thermo Fisher Scientific, Germany) as described by the manufacturer and eluted with 33 μL of de-ionised water.

This aptamer library selected for capacity to bind to 8mers was then used for positive selection with a pool of blood serum from late stage P301S mice (the pool is composed of 2.5 μL of each mouse (numbers 3, 7, 8, 9 and 10)) in a total volume of 50 μL 1X Selection Buffer (Table 1). This solution was incubated with the immobilization field for 30 minutes at RT. The remaining solution was recovered carefully and stored in a fresh tube. The immobilization field was washed twice with 50 μL of selection buffer with each wash being collected and pooled with the solution removed in the first step. This solution contains sequences that did not bind to the immobilization, presumably because they are bound to some moieties within the blood serum instead. This pooled solution was purified as described for the phase I step, eluted in 400 μL and subjected to PCR amplification for an appropriate number of cycles to create a clear band of approximately 5 ng of amplified DNA. The PCR amplification of the library was performed according to standard molecular biology protocols. Thermal cycling consisted of 1 cycle 95°C for 5 min and x cycles of 95°C for 10 s, 55°C for 15 s, 72°C for 30 s.

**Table 1:** Mice used for individual serum selection.

Mouse	Genotyping	Sex	Age at sampling
1	TG Late	F	10 MONTHS 17 DAYS
2	TG Late	F	10 MONTHS 17 DAYS
3	TG Late	F	11 MONTHS 21 DAYS
4	TG Late	F	11 MONTHS 21 DAYS
5	TG Late	F	11 MONTHS 21 DAYS
6	TG Late	F	11 MONTHS 21 DAYS
7	TG Late	F	11 MONTHS 21 DAYS
8	TG Late	F	11 MONTHS 21 DAYS
9	TG Late	F	10 MONTHS 17 DAYS
10	TG Late	F	10 MONTHS 17 DAYS
11	WT Late	F	10 MONTHS 5 DAYS
12	WT Late	F	10 MONTHS 5 DAYS
13	WT Late	F	10 MONTHS 5 DAYS
14	WT Late	F	10 MONTHS 5 DAYS
15	WT Late	F	10 MONTHS 5 DAYS
16	WT Late	F	10 MONTHS 5 DAYS

TG = transgenic, WT = wild type, Late = advance age, F = female.

Subsequent selection rounds were performed in the same manner with the exception that a pool of blood serum from wild type (wt) mice (the pool is composed of 2.5 μL of blood serum from five wt mice selected randomly) was added in the negative selection phase (phase I of FRELEX), from selection rounds 2 to 6, where we select for aptamers that exhibit the capacity to bind to the immobilization field. The inclusion of wt blood serum enables the selection against aptamers that bind to targets that are present within this material. This creates a contrast in which we are effectively selecting aptamers that bind to epitopes that are enriched in the late transgenic blood pool. This selection process was repeated for a total of five selection rounds. Aliquots from selection rounds 5 and 6 were analyzed by next generation sequence analysis (standard approach) by the Hospital for Sick Children (Toronto, Canada) using an Illumina HiSeq instrument. Sequence analysis was performed using NeoVentures Biotechnology Inc.’s proprietary software.

### Individual mouse blood serum selection

The aptamer library from selection round 5 was divided into 10 μL aliquots. Each aliquot was applied to blood serum from a single mouse from each of the mice listed in Table 1 for a single selection round (phase II of FRELEX only). Following selection, each library selected from each individual mouse was characterized by next generation sequencing (standard approach) with an Illumina HiSeq instrument at the Hospital for Sick Children in Toronto, Canada. Sequence analysis was performed using NeoVentures Biotechnology Inc.’s proprietary software.

## Results and discussion

**Fig 2:**
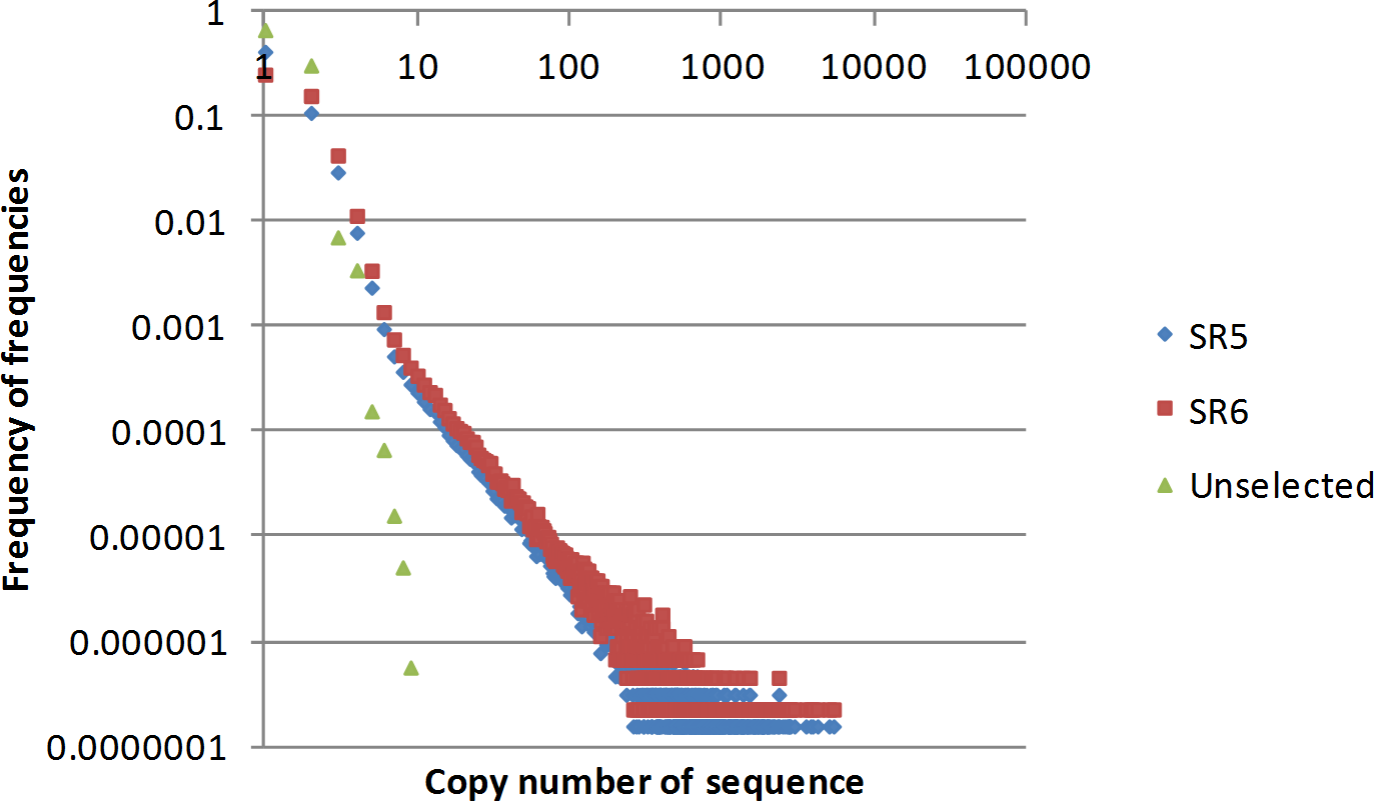
Sequence enrichment for the unselected library and selection round 5 and 6. On the x axis are the frequencies of each aptamer sequence observed by NGS analysis. On the y axis are the frequencies of the observation of these frequencies. SR5 = selection round 5, SR6 = selection round 6. Unselected = Purchased library from Trilink Biotechnologies.

We analyzed selection rounds 5 and 6 through next generation sequencing. The ideal selection round library to use for our purposes is a library in which the majority of the high copy number sequences are observed in a library from a subsequent round. We cannot determine changes in relative frequency of aptamers if we fail to observe the same sequences when applied to different samples. At the same time, we want to use a selection round at which this objective is first achieved. Aptamer evolution is a function of the individual binding affinity of each aptamer and the concentration of the epitope that it is binding to. Our target in this study, blood serum, is complex with differences in epitope frequency spanning many orders of magnitude. This means that with later rounds of selection we would have a greater opportunity to observe the same sequences from one selection round to the next, but that these sequences would be binding to only those targets that are present at higher frequency. From our own experience working with FRELEX in selection against blood serum we have learned that this inflection point is generally not before 5 rounds of selection. We observed a total of 6 375 637 sequences in selection library 5 and 4 396 923 sequences in selection library 6. These numbers include multiple copies of the same sequence. For comparison the copy number values for SR5 were normalized to the same frequency as those of SR6 by dividing the observed SR5 frequency by the ratio of the total number of sequences in SR5 by the total number in SR6. In Fig 2 we plot the copy number observed of sequences on the x axis by the frequency that this copy number was observed in the library The number of sequences that are represented by high copy numbers are less frequent as the selection progresses.

This provides an indication of the complexity of the library. It is clear in Fig 2 that library has decreased in complexity from the unselected library by selection round 5, and that this decrease in complexity changed very little from selection round 5 to selection round 6. For this reason, we decided to proceed with aliquots from selection round 5 for the individual mouse blood serum selection.

The higher the copy number of a sequence the more probable that we will observe it in both selection libraries. 242 615 sequences were observed at least twice in selection round 6. Of these, 61 933 sequences were also observed at least twice in selection round 5. This means that 180 682 sequences observed twice in selection round 6 were either not observed in selection round 5 or were observed only once.

Effective selection pressure results in a decrease in the total number of unique sequences and an increase in the copy number of the sequences that are binding to target molecules. Selection is driven by both the binding affinity of the aptamer to the target and the copy number of the target. Eventually over many rounds of selection those aptamers that bind with the highest affinity to the most abundant targets should dominate the library. Sequences that bind to targets with lower abundance will reach a peak in frequency and then begin to decrease in abundance. It should be kept in mind that throughout the selection process, counter selection is being performed against healthy blood serum. This means that the sequences increasing in abundance should primarily be binding to pathological epitopes. Sequences that bind with poor affinity to healthy epitopes will however be eliminated less efficiently from the selected library than sequences that bind with high affinity to healthy epitopes.

All of the top 1000 sequences in terms of copy number in selection round 5 were observed in selection round 6. We call such sequences observed in both selection rounds, "Aptamarkers". In the remainder of this paper we will confine our analysis to these top 1000 sequences.

We then took equal aliquots of selection round 5 and applied each aliquot to a single round of selection against a blood serum sample from an individual mouse. This was done with 16 different mice as listed in Table 1. Given that these selections are all parallel to each other in terms of the evolution of the selected library, differences in the relative frequency of aptamers from one mouse to another can be reasonably assumed to be caused by differences in the epitope abundance that these aptamers bind to.

Thus, we hypothesized that we would be able to use the relative abundance of aptamers to characterize the abundance of pathological epitopes in individual blood serum samples.

We first normalized aptamer frequencies by dividing the observed frequency for each of the 1000 most abundant aptamers in SR5 by the average frequency observed for these same 1000 aptamers in each mouse analyzed. This normalized data was then converted to a measure of variation from the average frequency observed for each aptamer by dividing the observed frequency from a single mouse by the average across all 16 mice and subtracting unity.

**Fig 3:**
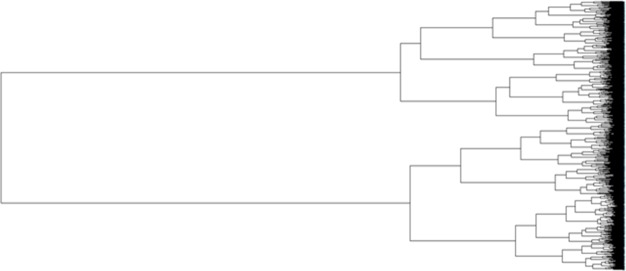
Phylogram of correlation among 1000 aptamers across 16 mice. Relative distances correlations for aptamer frequencies across the 16 mice. Distances are Euclidean distances between r^2^ values from the correlation matrix, and the hierarchal clustering was performed using the ward.D2 algorithm.

We are aware that certain aptamers may be binding to the same molecular entities in the blood. To characterize this, we performed covariance analysis of these 1000 Aptamarkers across the 16 mice. Correlation analysis was performed using the Pearson method. The correlation matrix was converted to a distance matrix using Euclidean distances. This was then sorted into clusters using the ward.D2 method (Fig 3).

**Fig 4:**
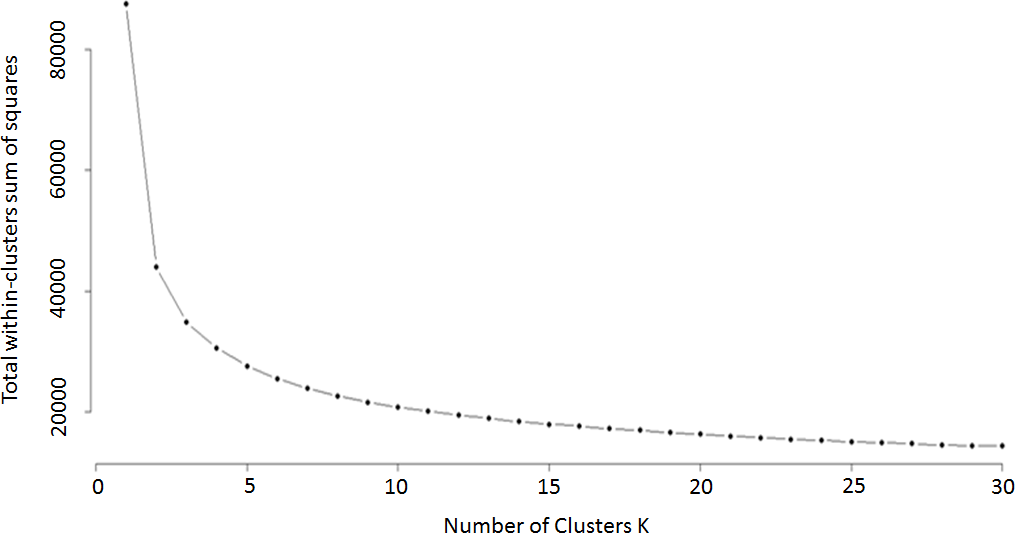
K analysis of optimum number of clusters. The x axis provides the number of clusters for each setting for k means analysis, the y axis provides the proportion of the variance explained by the number of clusters designated on the x axis.

Analysis of Fig 3 leaves us with the question as to what height we should cut off the number of clusters for analysis. First we looked at this question through k means analysis using the "elbow method" [8] (Fig 4). K means is a simple, unsupervised machine learning algorithm that starts with a random set of aptamer frequencies, and determines clusters based on the distance of other aptamer frequencies to these seed frequencies. An aptamer frequency is chosen as the center of each cluster (centroid) and the process is repeated based on this new set of aptamer frequencies. The process is repeated until there is no longer any improvement in the fit of aptamer frequencies to the clustering model. In this Elbow application, the k means process is repeated with the cluster number preset for each of the values represented on the x axis. The y axis denotes the amount of variation in the total data set explained by the clustering model. A sharp inflection in a line drawn across these points indicates the cutoff point for the number of relevant clusters to explain the data.

**Fig 5:**
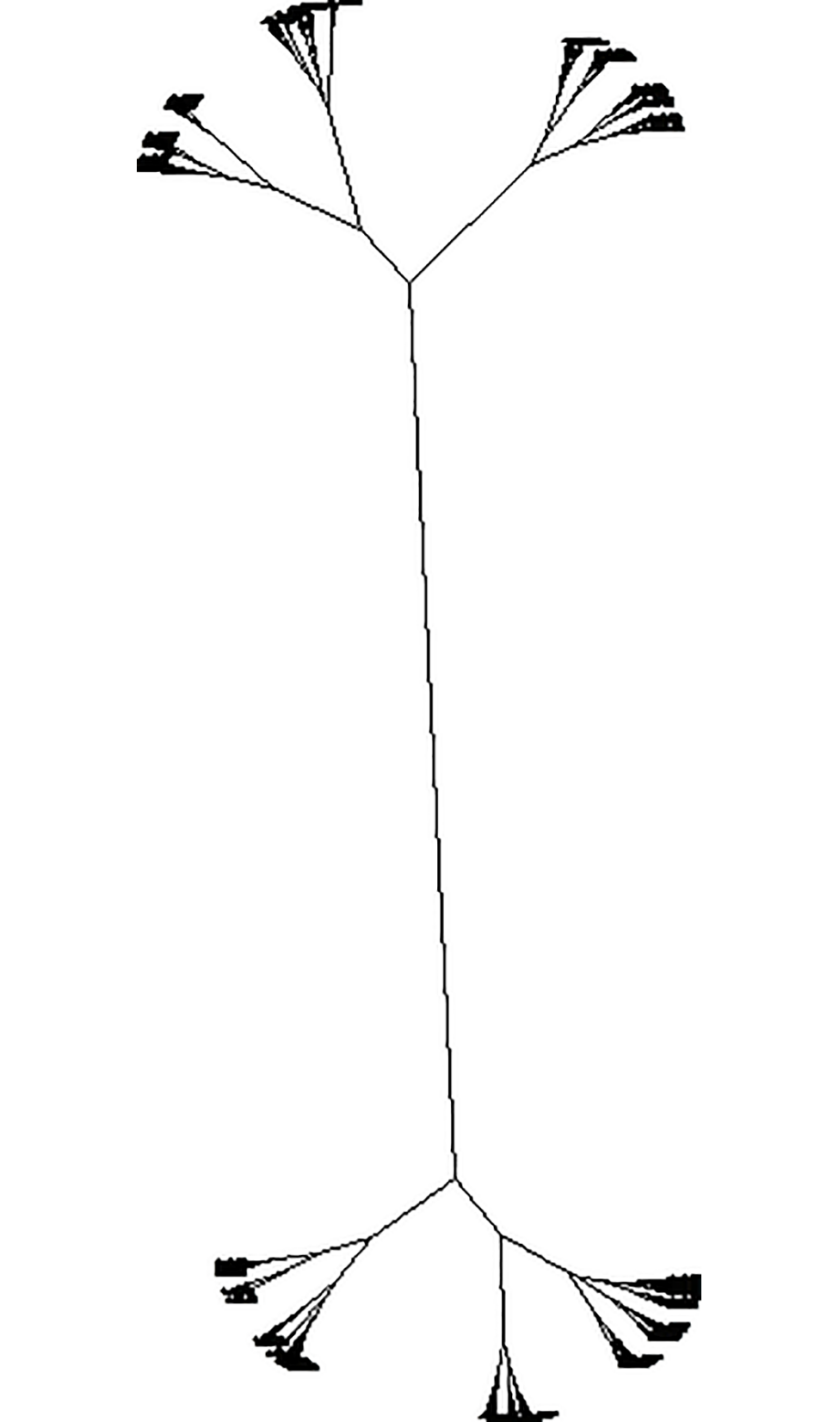
Unrooted phylogram of 1,000 aptamers across 16 mice defining clusters.

In this method we are looking for a clear bend in the graph, however here we have a relatively continuous curve. We found that observing the data with an unrooted tree phylogram to be a more effective means of determining cluster number (Fig 5).

**Fig 6:**
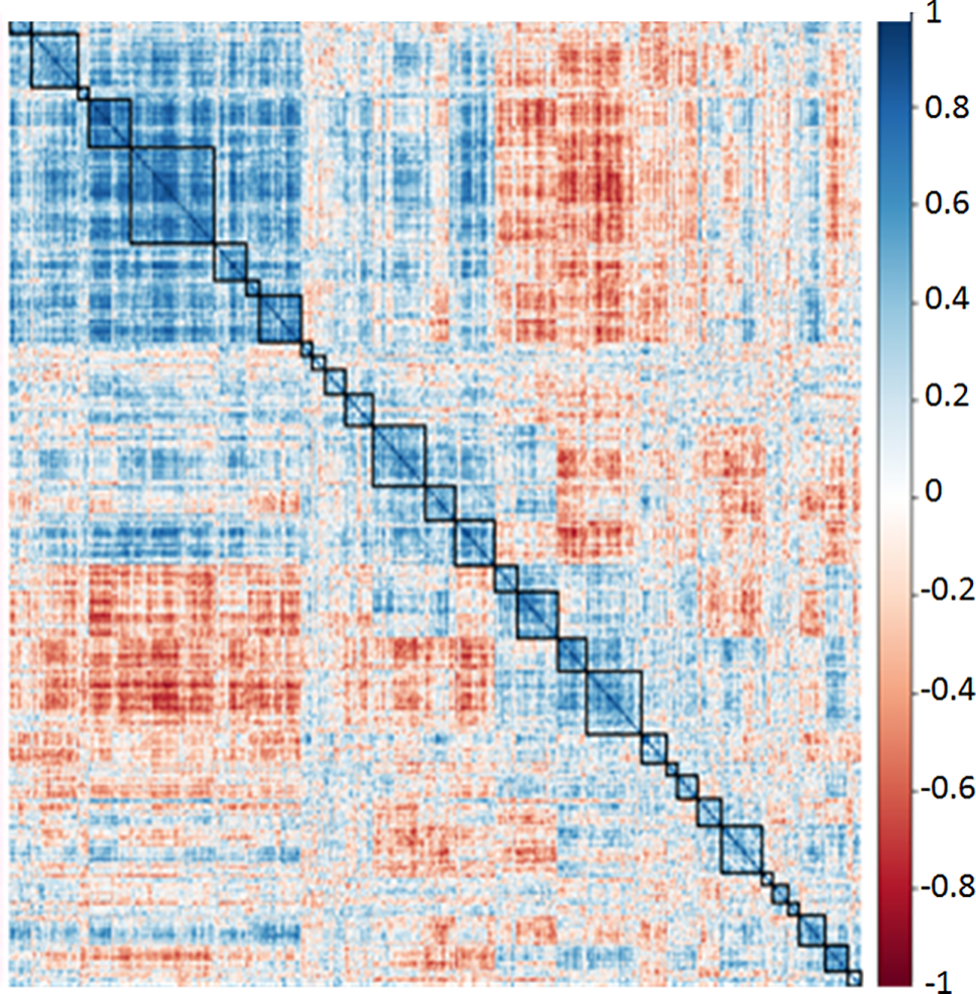
Correlation heat map of 1,000 aptamers across 16 mice. Based on the same distance matrix used for Figs 3 and 5, and on the ward.D2 algorithm for hierarchal clustering. Relative distances between aptamer frequencies are plotted in a colour gradient with dark blue being equal to +1, and dark red being equal to -1. Each row and each column correspond to a different specific aptamer.

This figure is based on the same distance matrix used for Fig 3. The assumption for plotting the distances is changed from ascribing a rooted origin point to not assuming any origin point at all. We then determine the number of meaningful clusters by counting the observable branches. Here we characterized 30 branches which we defined as clusters. There are undoubtedly meaningful subclusters within these branches, but 30 clusters provides us with a reasonable starting point for further analysis. These 30 clusters are labeled within squares in a correlation heat map of all 1,000 aptamers versus themselves (Fig 6).

Dark blue squares signify correlation coefficients approaching +1.0 while dark red squares signify correlation coefficients approaching -1.0. The thirty clusters defined are designated with black squares. The correlation heat map provides correlations for 1000 aptamers versus each other. As such there are 1000 columns and 1000 rows, with the positive correlation of each aptamer against itself being plotted on the diagonal. The half above the diagonal is a mirror image of the half below the diagonal. This plot was derived with the corrplot function in R.

**Fig 7:**
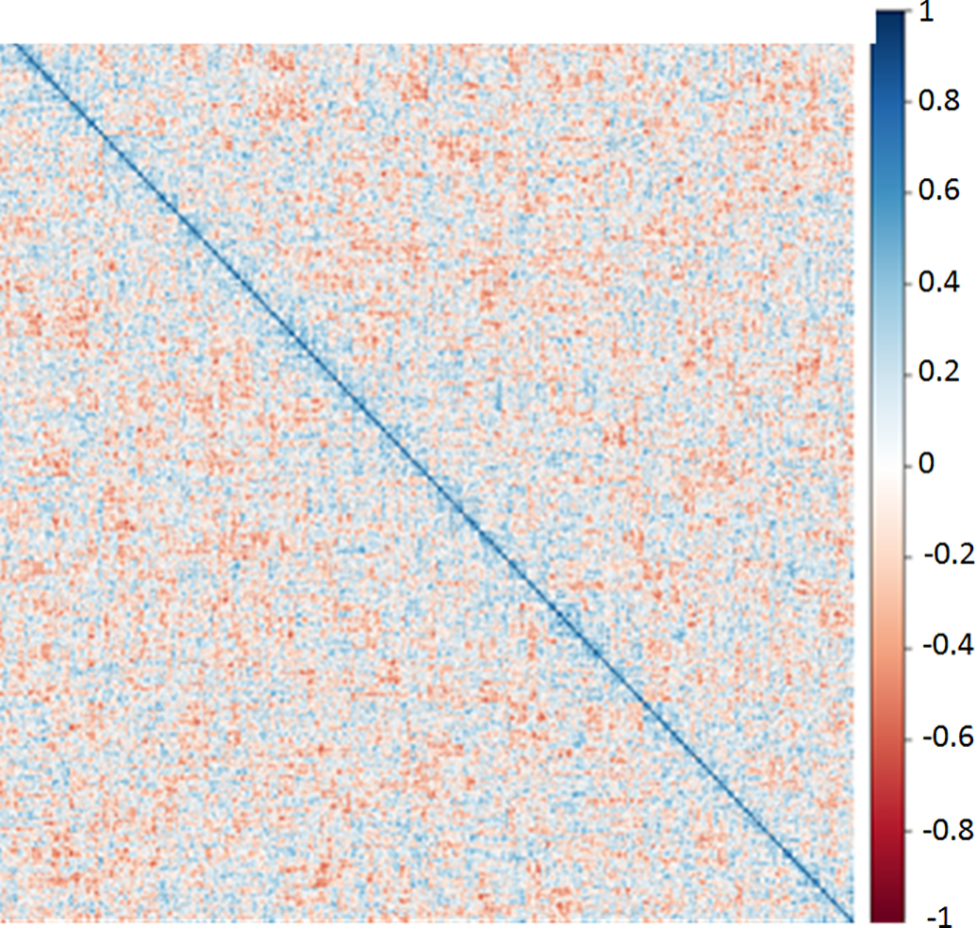
Analysis of randomly generated aptamer frequency data. The distance matrix based on randomly generated aptamer frequencies with the same mean and standard deviation as the observed aptamer frequencies plotted in Fig 6. Analytical approach was identical to that described for Fig 6.

We performed a simulated analysis with randomly generated data representing 1000 aptamers with equal mean and standard deviation to the observed data. In this case no significant clustering was observed (Fig 7).

**Fig 8:**
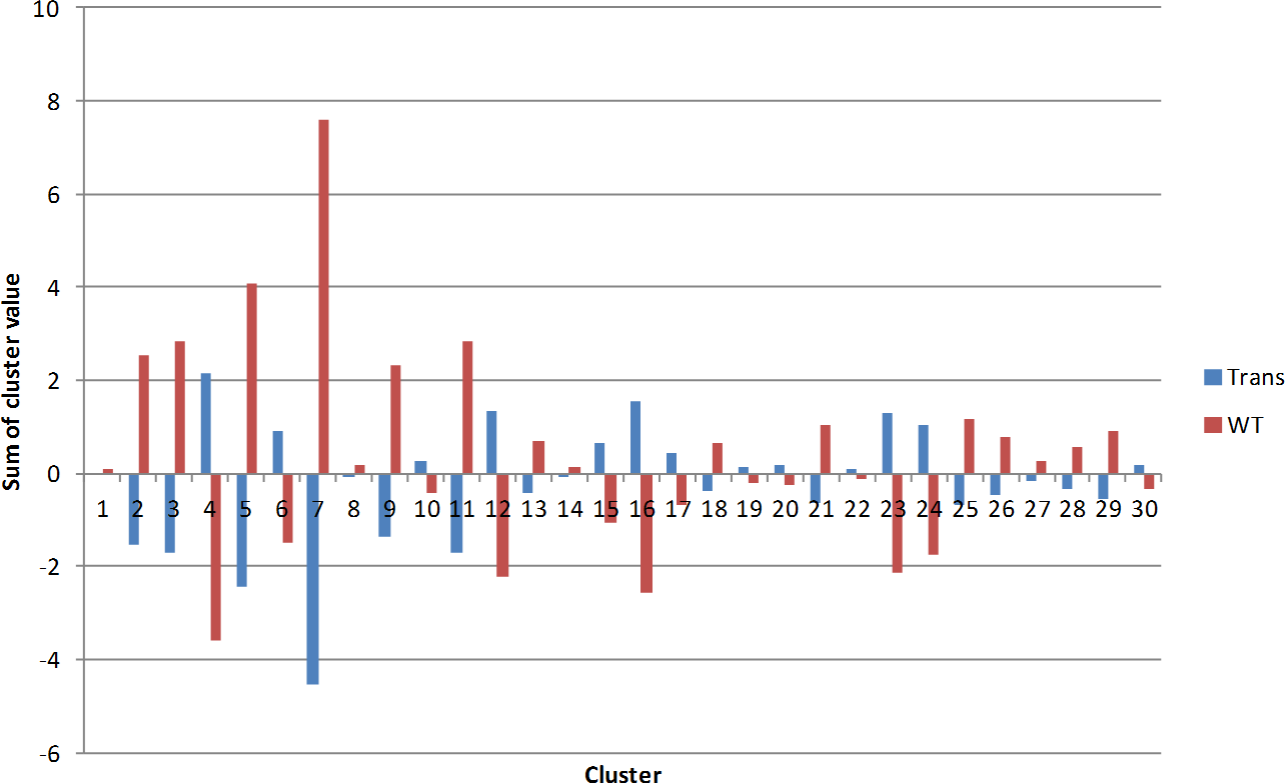
Average sum of frequencies within each cluster between transgenic and wild type mice. X axis represent each of the 30 clusters previously identified for this dataset. Sum of cluster value is the sum of the deviation from the average for each aptamer within a cluster. TRANS = transgenic mice, WT = wild type mice.

We interpret this result as meaning that the clusters in our observed data are biologically meaningful. We calculated the sum of the variation from average Aptamarker frequency within each of the 30 clusters for each of the 16 mice analyzed (Fig 8). Each aptamer frequency within each mouse was divided by the average frequency for that aptamer across all mice. This represents the individual deviation from average frequency. Then the sum of these deviations from average for each cluster of aptamers was determined for all the transgenic mice, and for the wild type mice.

**Fig 9:**
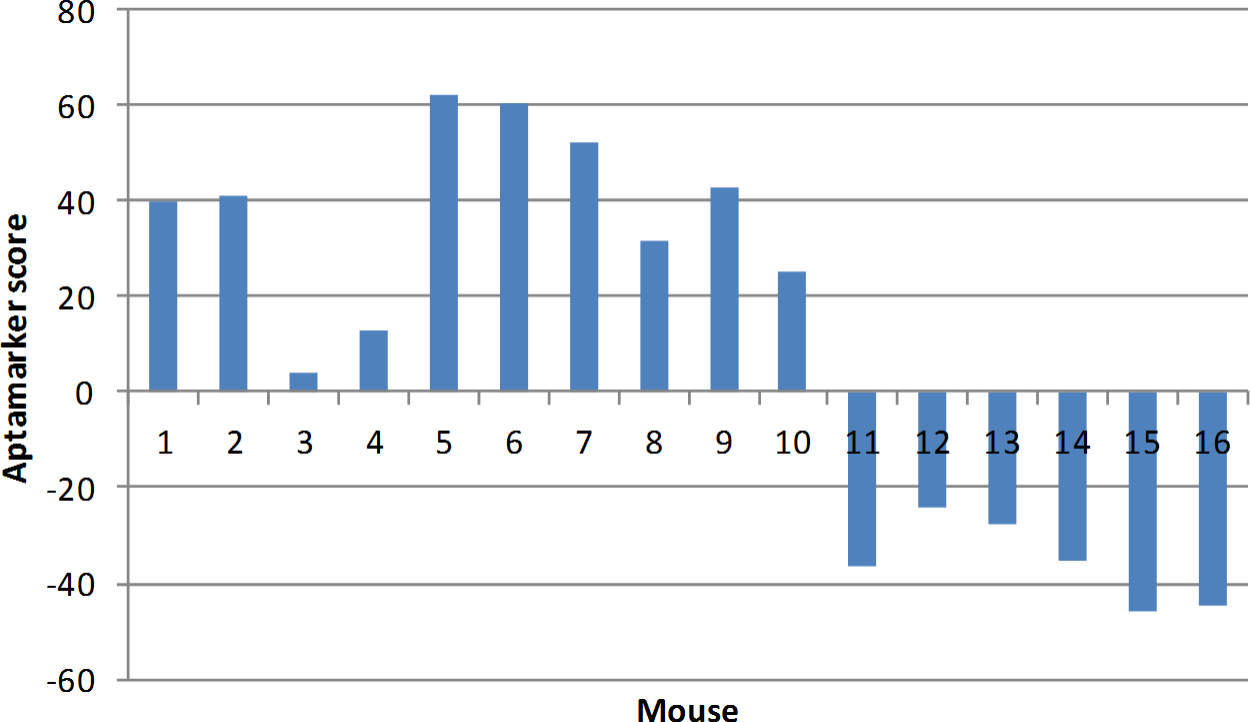
Individual comparison between transgenic and wild type mice. The X axis represents each individual mouse as noted in Table 1. Y axis provides the Aptamarker score according to the algorithm.

Based on the results observed in Fig 8 we devised a simple algorithm whereby we add the transgenic value for each cluster where it was larger than the wild type and subtract the value where the transgenic value was less than wild type. The sum of this algorithm is referred to as the Aptamarker score. An analysis of the individual mice shows that they are relatively consistent within each class (Fig 9).

**Fig 10:**
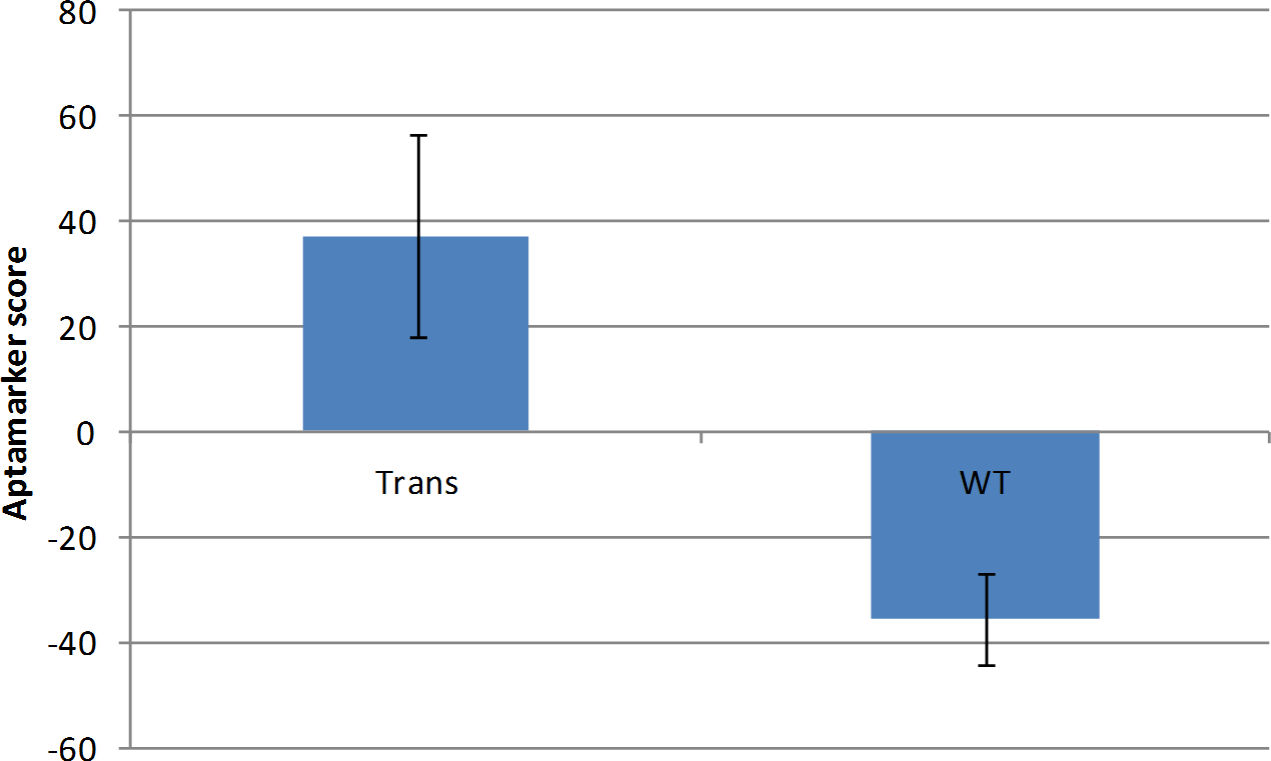
Overall comparison between transgenic and wild type mice. The averages across the transgenic and the wt mice in Fig 9 were used to generate the data presented in this figure.

Overall this led to a clear overall difference between the transgenic mice and the wild type mice (Fig 10) (P = 5.04E-07).

We have avoided a tautological approach to the application of the Aptamarker data to the differentiation of transgenic mice from wild type mice by using the data from all 1000 aptamers. An analysis of a subset of the clusters or choosing certain aptamers and not others would provide even greater separation between the types of mice. Such a choice of aptamers would be the basis of a trained Aptamarker set and the basis of a diagnostic platform. However, in this study we wished only to demonstrate that the differences between transgenic mice and wt mice could be observed with an analysis of the whole data set. It is interesting that the Aptamarker matrix as a whole is affected by the genotype of the mouse analyzed. This does not mean that all the Aptamarkers bind to meaningful biomarkers that are uniquely expressed in transgenic mice. It is clear that certain aptamers exhibited frequencies that were lower in the transgenic mice than in the wt. This can be explained if we assume that these aptamers were binding to healthy epitopes that remained in the selection, but were present at a lower abundance in the transgenic mice. It should be kept in mind that lower abundance does not necessarily reflect lower expression, Aptamarker frequencies are a function of the abundance of the epitope that they bind to relative to the abundance of all epitopes that all aptamers in the library bind to. The individual frequencies observed for each Aptamarker are a function of the evolution of the library as a whole. This is dependent on the relative frequency of the epitopes that the library binds to in each mouse, and the selection response of the aptamers in the reference library to these epitopes frequencies. Relative frequencies are meaningful only through the lens of the selected reference library.

Given the relatively low cost of parallel next generation sequence analysis it is not unreasonable to speculate that this approach could become the basis of a commercial diagnostic approach. The approach is agnostic in the sense that no information is provided as to the identity of the factors that are causing changes in the Aptamarker frequencies. This does not mean however, that significant correlations between the Aptamarker frequencies and phenotype diagnosis could not be meaningfully applied.
